# Epidemiologic and spatiotemporal trends of Zika Virus disease during the 2016 epidemic in Puerto Rico

**DOI:** 10.1371/journal.pntd.0008532

**Published:** 2020-09-21

**Authors:** Tyler M. Sharp, Talia M. Quandelacy, Laura E. Adams, Jomil Torres Aponte, Matthew J. Lozier, Kyle Ryff, Mitchelle Flores, Aidsa Rivera, Gilberto A. Santiago, Jorge L. Muñoz-Jordán, Luisa I. Alvarado, Vanessa Rivera-Amill, Myriam Garcia-Negrón, Stephen H. Waterman, Gabriela Paz-Bailey, Michael A. Johansson, Brenda Rivera-Garcia

**Affiliations:** 1 Dengue Branch, Centers for Disease Control and Prevention, San Juan, Puerto Rico; 2 US Public Health Service, Rockville, Maryland, United States of America; 3 Office of Epidemiology, Puerto Rico Department of Health, San Juan, Puerto Rico; 4 Biological and Chemical Emergencies Laboratory, Puerto Rico Department of Health, San Juan, Puerto Rico; 5 Ponce Health Sciences University, Ponce, Puerto Rico; Australian Red Cross Lifelood, AUSTRALIA

## Abstract

**Background:**

After Zika virus (ZIKV) emerged in the Americas, laboratory-based surveillance for arboviral diseases in Puerto Rico was adapted to include ZIKV disease.

**Methods and findings:**

Suspected cases of arboviral disease reported to Puerto Rico Department of Health were tested for evidence of infection with Zika, dengue, and chikungunya viruses by RT-PCR and IgM ELISA. To describe spatiotemporal trends among confirmed ZIKV disease cases, we analyzed the relationship between municipality-level socio-demographic, climatic, and spatial factors, and both time to detection of the first ZIKV disease case and the midpoint of the outbreak. During November 2015–December 2016, a total of 71,618 suspected arboviral disease cases were reported, of which 39,717 (55.5%; 1.1 cases per 100 residents) tested positive for ZIKV infection. The epidemic peaked in August 2016, when 71.5% of arboviral disease cases reported weekly tested positive for ZIKV infection. Incidence of ZIKV disease was highest among 20–29-year-olds (1.6 cases per 100 residents), and most (62.3%) cases were female. The most frequently reported symptoms were rash (83.0%), headache (64.6%), and myalgia (63.3%). Few patients were hospitalized (1.2%), and 13 (<0.1%) died. Early detection of ZIKV disease cases was associated with increased population size (log hazard ratio [HR]: -0.22 [95% confidence interval -0.29, -0.14]), eastern longitude (log HR: -1.04 [-1.17, -0.91]), and proximity to a city (spline estimated degrees of freedom [edf] = 2.0). Earlier midpoints of the outbreak were associated with northern latitude (log HR: -0.30 [-0.32, -0.29]), eastern longitude (spline edf = 6.5), and higher mean monthly temperature (log HR: -0.04 [-0.05, -0.03]). Higher incidence of ZIKV disease was associated with lower mean precipitation, but not socioeconomic factors.

**Conclusions:**

During the ZIKV epidemic in Puerto Rico, 1% of residents were reported to public health authorities and had laboratory evidence of ZIKV disease. Transmission was first detected in urban areas of eastern Puerto Rico, where transmission also peaked earlier. These trends suggest that ZIKV was first introduced to Puerto Rico in the east before disseminating throughout the island.

## Author summary

During epidemics of Zika virus disease in the Americas in 2015 and 2016, assessment of transmission dynamics was limited by inconsistent laboratory testing of patients with suspected Zika virus disease. This limitation was further complicated by co-circulation of dengue and chikungunya viruses, which cause illnesses clinically similar to Zika virus disease. In Puerto Rico, all reported suspect cases of arboviral disease were tested for Zika, dengue, and chikungunya virus infection throughout the epidemic, which allowed for fine-scale analysis of epidemiologic and spatiotemporal trends. In total, 39,717 cases of Zika virus disease were detected, or roughly 1% of all residents of Puerto Rico. Young adults and females were most affected. Disease was mostly mild, as only 1% of cases were hospitalized. Thirteen patients with Zika virus disease died, most of whom had Guillain-Barré syndrome or severe underlying illnesses. Early detection of Zika virus disease cases was associated with more populated areas of eastern Puerto Rico, where early detection of peak case numbers also occurred, particularly in warmer areas. These trends suggest that, in contrast to prior epidemics of dengue and chikungunya that started in the San Juan metropolitan region, the Zika virus epidemic appears to have begun in eastern Puerto Rico.

## Introduction

Zika virus (ZIKV) was first associated with human disease in 1953 [1]; however, data regarding the characteristics of ZIKV disease were limited by only 13 cases being documented over the following 50 years, and reported outbreaks were mainly limited to Pacific islands [2]. During one such outbreak in Yap State, Micronesia in 2007, ~73% of residents were infected, of whom ~19% developed symptoms including fever, rash, arthralgia, and conjunctivitis, and ~2% presented for clinical care [3]. Hence, when ZIKV spread to the Americas during 2014–2015 [2], there was a paucity of population-level data on spatiotemporal factors associated with spread and risk factors for developing ZIKV disease. Such factors typically inform approaches to disease surveillance and case reporting, which are used to direct public health resources to affected areas.

Like ZIKV, dengue (DENV) and chikungunya (CHIKV) viruses are transmitted by select *Aedes* species mosquitos and cause an acute illness that often includes fever, rash, myalgia, and arthralgia [4, 5]. Because many areas of the Americas in which ZIKV recently emerged had ongoing circulation of DENV and CHIKV, laboratory-based diagnostic testing was needed to reliably differentiate patients with ZIKV disease from those with dengue or chikungunya [6–8]. However, resources for diagnostic testing are often limited during epidemics, and complete diagnostic testing for ZIKV, DENV, and CHIKV infection for all suspected cases is typically not available, thus complicating analysis of large-scale epidemiologic trends. Of note, travel importations, temperature, and socio-economic factors have been associated with the introduction and spread of ZIKV throughout the Americas [9]. Similarly, models of global ZIKV transmission found that climatic variations (e.g., increasing temperatures) due to El Niño were associated with increased risk and subsequent spread of ZIKV in South America [10]. On smaller geographical scales, however, the drivers of spread are not well understood, but are needed to improve the understanding of the spatial and temporal characteristics of ZIKV spread to thereby inform mitigation measures during future outbreaks. Identification of these determinants would benefit from large datasets in which patients were routinely tested to confirm ZIKV infection and rule out infection with DENV and CHIKV.

Surveillance for dengue has operated in the Caribbean U.S. territory of Puerto Rico since the 1960s [11] and was expanded to include chikungunya in 2014 [12]. Following detection of ZIKV transmission in the Americas in 2015 [13], Puerto Rico Department of Health (PRDH) initiated laboratory-based surveillance for ZIKV disease through the existing arboviral diseases surveillance system [14]. Although pregnant women and hospitalized patients were prioritized, diagnostic testing was performed on all reported cases of suspected arboviral disease [14]. In addition to surveillance for arboviral diseases, enhanced surveillance was conducted for disease complications associated with ZIKV infection, including Guillain-Barré syndrome (GBS) and severe thrombocytopenia [15, 16]. Surveillance for congenital Zika syndrome was also implemented, the initial findings of which have been reported elsewhere [17].

In this analysis, we describe the epidemiologic trends of ZIKV disease cases reported during the epidemic in Puerto Rico. We also investigate the spatiotemporal patterns of ZIKV disease cases by analyzing the relationship between spatiotemporal indicators of epidemic progression with geographic, demographic, and climatic characteristics using parametric survival and regression analyses.

## Methods

### Investigation design and ethics statement

A retrospective analysis of suspected ZIKV disease cases reported to PRDH was performed to: 1) describe the epidemiology of the 2015–2016 epidemic of ZIKV disease; and, 2) analyze the spatiotemporal trends of the epidemic by municipality. The investigation protocol was approved by the Centers for Disease Control and Prevention (CDC) Institutional Review Board.

### Case reporting

Patients with suspected ZIKV disease were first reported in July 2015. Following detection of the first laboratory-confirmed case in late November 2015, routine retrospective testing of pathogen-negative specimens was performed for patients with reported illness onset in November and December 2015. Beginning in January 2016, all suspected arboviral disease cases reported to PRDH were tested for evidence of infection with ZIKV, DENV, and CHIKV. Specimens were submitted by healthcare providers along with an Arbovirus Case Investigation Form (ACIF) (http://www.salud.gov.pr/Sobre-tu-Salud/Documents/ACIF_FillableFields_Spanish_032916.pdf), and tested by PRDH or CDC Dengue Branch, both located in San Juan, Puerto Rico. Data were also included from cases reported from the Sentinel Enhanced Dengue Surveillance System (SEDSS), a facility-based acute febrile illnesses surveillance system that operates at two healthcare facilities in Ponce, Puerto Rico [18].

Enhanced GBS surveillance and retrospective case finding for ZIKV-associated severe thrombocytopenia in Puerto Rico have been previously described [15, 16, 19–21]. Medical records of reported GBS cases were retrospectively reviewed to determine if cases met the Brighton Collaboration Criteria for GBS [22]. Suspected GBS cases that met Brighton level I–III criteria and had a clinical specimen submitted for diagnostic testing were included in this analysis. Medical records of patients with ZIKV infection and reported thrombocytopenia were reviewed to identify cases of severe thrombocytopenia [16, 21]. Pregnant women reported with suspected arboviral disease are included in this analysis, whereas asymptomatic pregnant women screened for ZIKV infection were excluded from this analysis and will be reported elsewhere.

### Diagnostic testing

Suspected arboviral disease cases reported to PRDH were tested by PRDH or CDC according to status of pregnancy and day post-illness onset of serum specimen collection (S1 Fig). Serum specimens from non-pregnant case-patients collected within seven days of illness onset were tested with the CDC Trioplex real-time reverse transcriptase-polymerase chain reaction (RT-PCR) assay to detect ZIKV, DENV, and CHIKV nucleic acid [23]. Serum specimens collected four or more days post-illness onset were tested by anti-ZIKV IgM antibody capture enzyme-linked immunosorbent assay (MAC ELISA) [24], as well as by anti-DENV MAC ELISA (InBios International, Inc., Seattle, WA) as resources allowed. Because viremia is extended in pregnant women [25, 26] and ZIKV RNA is detectable in urine [27], both serum and urine specimens from symptomatic pregnant women were tested by RT-PCR regardless of day post-illness onset of specimen collection. All serum specimens from symptomatic pregnant women that tested negative by RT-PCR were tested by anti-ZIKV MAC ELISA, and those that tested positive or equivocal by anti-ZIKV MAC ELISA were prioritized for further testing by anti-DENV MAC ELISA. Specimens positive for detection of DENV nucleic acid by Trioplex RT-PCR assay were further tested by the CDC DENV-type-specific real-time RT-PCR assay [28] to identify the infecting DENV type. Molecular and serologic diagnostic testing for ZIKV, DENV, and CHIKV infection in patients detected via SEDSS was performed as previously described [18, 29].

### Definitions

A suspected arboviral disease case was any patient reported to PRDH during November 2015–December 2016 with a completed ACIF, regardless of clinical suspicion for dengue, chikungunya, or ZIKV disease. Confirmed ZIKV disease cases were defined by detection of ZIKV nucleic acid by RT-PCR, and probable ZIKV disease cases by detection of anti-ZIKV IgM antibody by ELISA regardless of if testing by anti-DENV IgM ELISA was performed or the result. Confirmed dengue cases were defined by detection of DENV nucleic acid by RT-PCR, and probable dengue cases by detection of anti-DENV IgM antibody by ELISA with negative anti-ZIKV IgM ELISA testing. Confirmed chikungunya cases were defined by detection of CHIKV nucleic acid by RT-PCR, and probable chikungunya cases by detection of anti-CHIKV IgM antibody by ELISA with negative by anti-ZIKV and -DENV IgM ELISAs.

For cases in which no date of illness onset was reported (n = 5,217; 8%), date of illness onset was imputed to be four days prior to specimen collection to ensure specimens received testing by both RT-PCR and IgM. As no arboviral co-infections were identified, cases were only counted under a single case definition.

### Spatiotemporal analyses

The 2016 US Census Bureau population estimate for Puerto Rico (total population: 3,406,495) and municipal population estimates (mean population 36,673 [interquartile range (IQR): 23,163–49,361) were used to calculate population densities and annual incidence rates for each of the 78 municipalities [30]. Municipality-specific temperatures (overall annual mean: 25°C) and precipitation (overall annual mean: 1,685 mm/year) were estimated from a model based on weather station data collected during 1986–2000 [31]. Altitude (overall mean: 64 meters) was extracted from United States Geological Survey data [32]. For each municipality, we estimated travel time from the centroid of the municipality to the nearest city, defined and referred throughout the remainder of the text as a municipality comprised of ≥50,000 residents [33]. Fifteen municipalities had cities that met this criterion, most of which surrounded either San Juan (northern region) or Ponce (southern region). Similarly, the distance (in kilometers [km]) to San Juan was estimated by using the latitude and longitude of a municipality’s centroid and the latitude and longitude of San Juan’s centroid to obtain the geodesic distance between them. Socio-economic data for each municipality was collected from the US Census Bureau’s 2015 American Community Survey [34].

For each municipality, we identified two outcomes: the week of the first confirmed ZIKV disease case, and the midpoint of the outbreak, defined as the week when half of the cumulative confirmed ZIKV disease cases was reached in 2016. This midpoint provides a more robust indicator of the municipality-level peak of incidence than the week with the most confirmed cases due to the sporadic reporting of cases in less populated municipalities. The time resolution of the two analyses was at the week-level. Using a time-to-event regression model, we analyzed the association between each outcome and the following nine municipality-specific determinants: population size, latitude, longitude, mean altitude (meters), travel time (minutes) to a city, distance to San Juan (kilometers), mean temperature (C), mean monthly precipitation (millimeters), and inclusion in the SEDSS catchment area. All variables except the categorical SEDSS catchment variable were continuous variables and modeled linearly or as a non-linear spline term. We first assessed four parametric survival models: Weibull, exponential, log-normal, and Gaussian. The Weibull model had the lowest Akaike’s Information Criteria (AIC) estimate, a measure of model fit for time to the first confirmed case (AIC: 496) and had similar AIC as a Gaussian model (AIC: 446 vs 445) for the time to midpoint of outbreak model. Therefore, we selected the Weibull model for all time-to-event analyses (S1 Table). We then performed univariate risk factor analyses for each outcome by assessing the strength of association between each of the nine individual characteristics and the outcomes. In multivariate analyses, we evaluated combinations of risk factors, selecting covariates based on statistical significance of coefficients, and the coefficient of correlation (R^2^) of model estimates to the observed data. We also assessed the non-linear relationship between risk factors and our outcomes using generalized additive models to estimate penalized-spline terms (gamlss package in R). We report the adjusted log hazard estimates (HR) and 95% confidence intervals (CI).

We also examined the relationship between the outcome of total ZIKV disease cases in 2016 (i.e., incident ZIKV disease cases) and socioeconomic, geographic, and climatic factors. Using univariate and then multivariable negative-binomial regression, we analyzed the association between confirmed ZIKV disease cases offset by the population size and the following twelve municipality-specific socio-economic, geographic, and climatic characteristics: percent employed (%), average time to work (minutes), median household income (in US dollars), average household size, median household age (years), population density, latitude, longitude, travel time (minutes) to a city, mean temperature (C), mean precipitation (millimeters), and inclusion in the SEDSS catchment area.

For all analyses, we fit the final models excluding observations for Vieques and Culebra, two island municipalities with small, isolated populations that could have distinct patterns of spread. Excluding these two municipalities had little effect on overall model fit; therefore, we included them in all analyses.

### Investigation data

De-identified data from the investigation reported herein are available in Supporting Information.

## Results

### Case detection

A total of 71,618 suspected arboviral disease cases were reported during November 1, 2015 through December 31, 2016 (20.3 suspected cases per 1,000 population). Of these, 64,760 (90.4%) cases were reported via passive surveillance for arboviral disease, 6,669 (9.3%) via the sentinel surveillance system in Ponce (SEDSS), and 189 (0.3%) via enhanced surveillance for GBS.

Of the suspected arboviral disease cases, 39,717 (55.5%, 11.3 cases per 1,000 population) were ZIKV disease cases: 36,390 (91.9%) were confirmed ZIKV disease cases, including 36,177 that were positive by RT-PCR in serum, 212 in urine only, and one in CSF only; and 3,327 (8.3%) were probable ZIKV disease cases, including 344 (10.3%) that tested positive by both anti-ZIKV and anti-DENV IgM ELISA.

Among the 31,681 (44.2%) suspected arboviral disease cases that tested negative for ZIKV infection, 198 dengue cases were identified, of which 91 (46.0%) were confirmed dengue cases and 107 (54.0%) were probable dengue cases. Among 30 cases in which the infecting DENV was identified, 25 were DENV-2, three were DENV-4, and one each was DENV-1 and DENV-3. In addition, 149 chikungunya cases were detected: 77 (51.7%) were confirmed chikungunya cases, and 72 (48.3%) were probable chikungunya cases. No cases were detected with DENV, CHIKV, or ZIKV co-infection. Diagnostic testing was unable to be performed for 177 (0.3%) suspected arboviral disease cases.

### Epidemiologic trends

**Fig 1:**
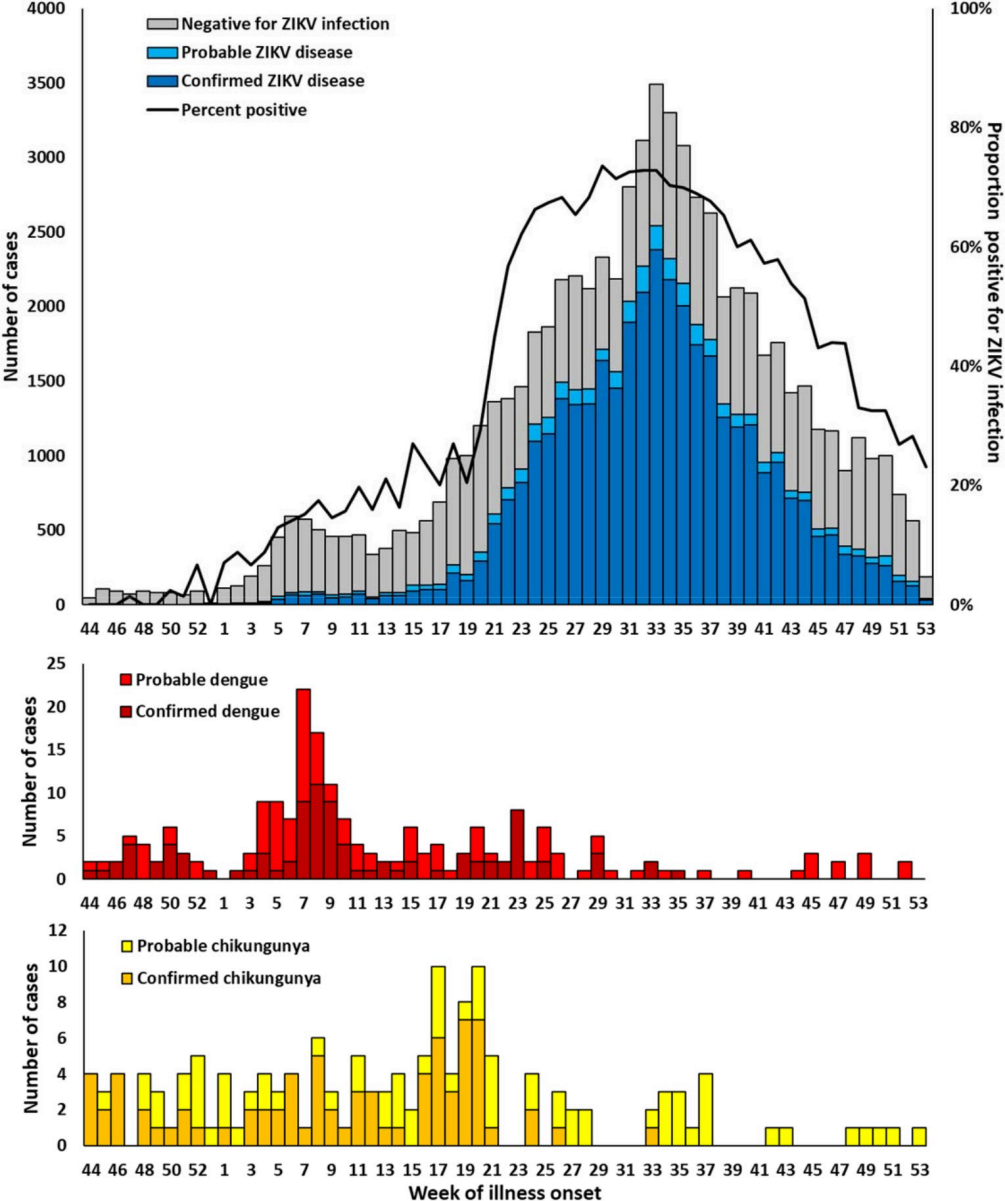
Reported arboviral disease cases by diagnostic test result and reported week of illness onset, November 1, 2015–December 31, 2016 (N = 71,618). Top: Confirmed and probable Zika virus (ZIKV) disease cases (n = 39,717), and cases negative for ZIKV infection (gray bars; n = 31,681); Middle: Confirmed and probable dengue cases (n = 198); Bottom: Confirmed and probable chikungunya cases (n = 149).

The first ZIKV disease case detected had reported illness onset in late November 2015 (Fig 1). The proportion of suspected arboviral cases that tested positive for ZIKV infection increased steadily during December 2015 and the first quarter of 2016. The number of ZIKV disease cases detected each week remained roughly stable until the second quarter of 2016 when case numbers and rates of specimen positivity both sharply increased. The peak number of ZIKV disease cases occurred during week 33 (mid-August) when 2,542 ZIKV disease cases were detected. During the four peak weeks of the epidemic, 70–74% of suspected arboviral disease cases tested positive for ZIKV infection. The weekly number of ZIKV disease cases decreased steadily thereafter through the end 2016. The proportion of suspected cases testing positive for ZIKV disease was higher among non-pregnant as compared to pregnant case-patients throughout the year (57.5% vs. 31.5%, respectively; chi-squared, *P* < 0.001).

Dengue and chikungunya cases were detected in most weeks for the first half of 2016 but were less frequent in the second half of the year despite increased suspected arboviral case numbers, co-incident with increased identification of ZIKV disease cases. Overall, weekly numbers of dengue and chikungunya cases detected was low compared to both ZIKV disease cases and historic weekly dengue and chikungunya case numbers.[12, 35]

**Fig 2:**
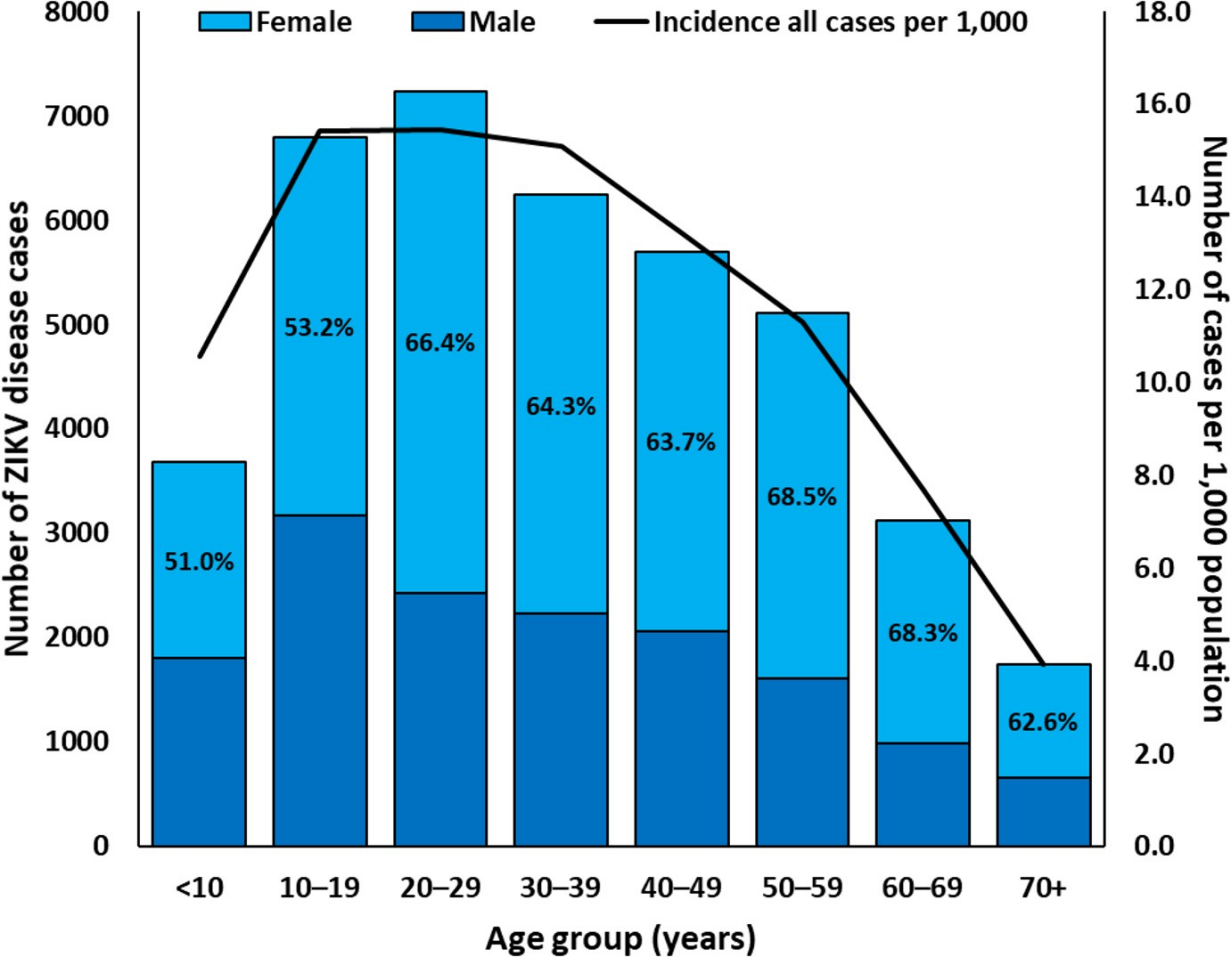
Zika virus disease cases by sex and number per 1,000 population by age group (N = 39,637*), Puerto Rico, November 1, 2015–December 31, 2016. Percentages in black indicate the proportion of female cases by age group. *Neither age nor date of birth were available for 80 cases.

ZIKV disease cases were identified in all age groups (Fig 2). Incidence of ZIKV disease was highest among individuals aged 20–29 years (15.5 cases per 1,000 individuals) and lowest among individuals aged ≥70 years (3.9 cases per 1,000 individuals). Most (62.3%) ZIKV disease cases were female. Although the distribution of cases by sex was similar among ZIKV disease case-patients aged <20 years (i.e., 52.4% female), nearly two-thirds (65.8%) of ZIKV disease cases aged ≥20 years were female. This disparity was greatest among ZIKV disease case-patients aged 50–59 years (68.5% female).

**Fig 3:**
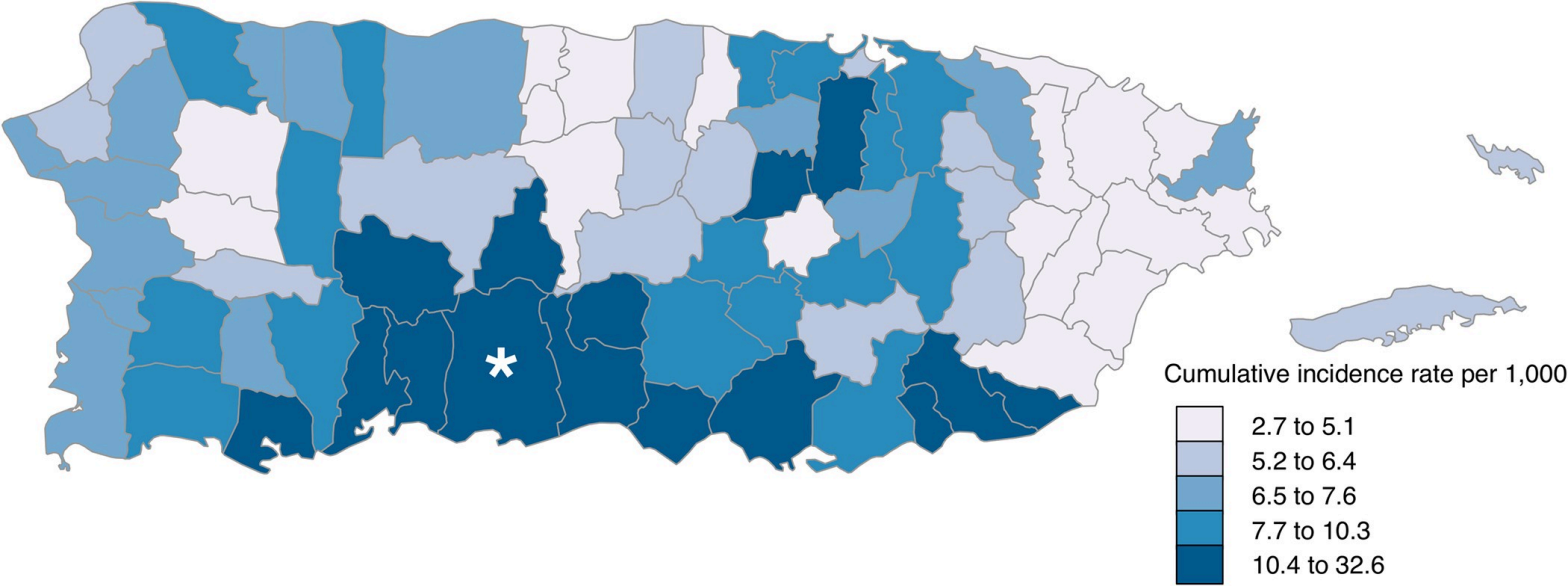
Incidence of laboratory-positive Zika virus disease cases (N = 39,578^†^) per 1,000 residents by municipality of residence, Puerto Rico, November 1, 2015–December 31, 2016. *Indicates the location of sites for enhanced surveillance for arboviral diseases ^†^Municipality of residence was not available for 139 cases.

ZIKV disease cases were detected among residents of all 78 municipalities of Puerto Rico (Fig 3). Incidence was highest among residents of Peñuelas (32.6 cases per 1,000 residents) in the coastal south, and lowest among residents of Ciales (2.7 cases per 1,000 residents) in the mountainous center of the island. Median incidence among all municipalities was 7.0 cases per 1,000 residents.

### Clinical characteristics

The most common signs and symptoms reported among ZIKV disease patients were rash (83.0%), headache (64.6%), myalgia (63.3%), and arthralgia (59.7%) (Table 1). Fever was reported for roughly half (51.9%) of ZIKV disease case-patients, and conjunctivitis by comparatively few (17.9%). Hospitalization was an infrequent outcome among both ZIKV disease case-patients (1.2%) and laboratory-negative case-patients (4.5%), as was death (0.03% and 0.11%, respectively).

**Table 1:** Demographic and clinical characteristics of reported arboviral disease cases and Zika virus diagnostic test results, Puerto Rico, November 1, 2015–December 31, 2016.

	All reported cases (N = 71,618)	Zika virus disease cases (n = 39,717)	Laboratory-negative cases* (N = 31,377)
**Demographic characteristics**			
Age, median (range)	30.6 years (0 days–100 years)	33.4 years (0 days–100 years)	27.5 years (0 days–100 years)
Female sex, n (%)	44,793 (62.5)	24,715 (62.2)	19,764 (63.0)
Pregnant, n (%)	5,767 (8.1)	1,816 (4.6)	3,910 (12.5)
Reported travel outside of Puerto Rico in the 14 days before illness onset, n (%)	1,661 (2.3)	866 (2.2)	782 (2.5)
**Clinical characteristics, n (%)**			
Signs and symptoms			
Rash	44,229 (61.8)	32,963 (83.0)	11,085 (35.3)
Headache	44,671 (62.4)	25,672 (64.6)	18,706 (59.6)
Myalgia	42,321 (59.1)	25,135 (63.3)	16,917 (53.9)
Arthralgia	38,822 (54.2)	23,713 (59.7)	14,866 (47.4)
Eye pain	33,872 (47.3)	20,939 (52.7)	12,707 (40.5)
Fever**	39,411 (55.0)	20,589 (51.8)	18,483 (58.9)
Chills	31,256 (43.6)	16,755 (42.2)	14,247 (45.5)
Sore throat	23,096 (32.3)	11,467 (28.9)	11,465 (36.5)
Nasal congestion	19,832 (27.7)	7,239 (18.2)	12,421 (39.6)
Cough	19,672 (27.5)	6,894 (17.4)	12,586 (40.1)
Conjunctivitis	12,589 (17.6)	7,096 (17.9)	5,398 (17.2)
Diarrhea	11,880 (16.6)	5,977 (15.1)	5,777 (18.4)
Nausea/vomiting	13,929 (19.5)	5,266 (13.3)	8,493 (27.1)
Abdominal pain	12,538 (17.5)	5,405 (13.6)	7,006 (22.3)
Anorexia	10,563 (14.8)	4,134 (10.4)	6,292 (20.1)
Lethargy	8,529 (11.9)	2,958 (7.5)	5,448 (17.4)
Jaundice	959 (1.3)	524 (1.3)	428 (1.4)
Mucosal bleeding***	876 (1.2)	327 (0.8)	537 (1.7)
Seizures	310 (0.4)	114 (0.3)	189 (0.6)
Clinical syndromes			
Guillain-Barré syndrome	107 (0.1)	71 (0.2)	36 (0.1)
Severe thrombocytopenia	NA	12 (0.03)	NA
Clinical course			
Hospitalized	1,921 (2.7)	472 (1.2)	1,400 (4.5)
Fatal outcome	48 (0.1)	13 (<0.1)	34 (0.1)

*does not include 198 dengue cases, 149 chikungunya cases, and 177 cases for which diagnostic testing was unable to be performed

**current or in prior 7 days

***epistaxis, gingival bleed, hematemesis, melena, hematochezia, menorrhagia, or hematuria (>5 red blood cells per high powered field)

ZIKV disease cases were identified among 1,816 symptomatic pregnant women, including 1,556 (85.6%) that tested positive by RT-PCR. Enhanced surveillance detected 71 confirmed GBS cases with evidence of ZIKV infection [15]. Retrospective case finding identified 12 cases of severe thrombocytopenia among patients with ZIKV infection [16].

Among 13 ZIKV disease case-patients that experienced fatal outcome, two died following complications of Guillain-Barre syndrome [15, 36], one from severe thrombocytopenia [21], and another with *Leptospira spp*. bacteria co-infection [37]. Two additional fatal cases had suspected Guillain-Barre syndrome but lacked sufficient clinical findings to meet the GBS clinical case criteria. The remaining seven fatal cases were aged ≥65 years and had severe underlying co-morbidities including congestive heart failure, bacterial sepsis, and nosocomial infections.

### Spatiotemporal trends

We analyzed socio-demographic, climatic, and spatial factors associated with the 78 municipalities in Puerto Rico to describe spatiotemporal trends among the 36,390 confirmed ZIKV disease cases. At the municipality level, earlier reporting of the initial confirmed case was significantly associated with: increased population size (log hazard ratio [HR]: -0.22, 95% CI: -0.29, -0.14), eastern longitude (log HR: -1.04, 95% CI: -1.17, -0.91), residence in Ponce (where SEDSS operates) (log HR: -0.78, 95% CI: -1.18, -0.38) (Table 2), and proximity to a city (spline with 2.0 estimated degrees of freedom [edf]) (S2A Fig). We compared this model to one with population size, latitude, and longitude to assess model fit without the distance indicator (i.e., travel time). This simpler model captured the first reported cases in the island municipalities (Vieques and Culebra) better (AIC: 451 vs. 457), but predicted initial cases later in western municipalities and had lower overall accuracy (R^2^ = 0.38 vs. 0.41).

**Table 2:** Demographic, climatic, and spatial factors associated with estimated log hazard ratio and 95% confidence intervals of week of first confirmed Zika virus disease case and midpoint of the Zika virus outbreak by municipality, Puerto Rico, November 1, 2015–December 31, 2016.

Model outcome	Time to first confirmed case						Time to midpoint of outbreak					
Model type	Univariate models			Final model			Univariate models			Final model		
Variable	Estimate (95% CI)	AIC	R^2^	Estimate (95% CI)	AIC	R^2^	Estimate (95% CI)	AIC	R^2^	Estimate (95% CI)	AIC	R^2^
Log 2016 population	-0.52 (-0. 77, -0.28)	482	0.05	-0.22 (-0.29, -0.14)	451	0.41	-0.08 (-0.11, -0.05)	425	0.13		374	0.58
Longitude (in degrees)	-0.72 (-1.03, -0.41)	480	0.21	-1.04 (-1.17, -0.91)			-0.05 (-0.10, 0.01)	443	0.22	* 6.5 edf		
Latitude (in degrees)	0.72 (-0.44, 1.87)	497	0.02				-0.32 (-0.48, -0.16)	435	0.08	-0.27 (-0.29, -0.25)		
Mean altitude	0.001 (0.000, 0.002)	494	0.08				0.00 (0.00, 0.00)	438	0.21			
Travel time (minutes) to city	0.006 (0.000, 0.012)	492	0.13	* 2.0 edf			0.001 (0.000, 0.002)	439	0.06			
Distance (in Km) to San Juan	0.009 (0.004, 0.014)	486	0.13				0.001 (0.001, 0.002)	435	0.21			
Mean monthly temperature (C)	-0.19 (-0.33, 0.05)	490	0.13				-0.03 (-0.05, -0.01)	437	0.21	-0.04 (-0.05, -0.03)		
Mean monthly precipitation (mm)	0.000 (0.000, 0.001)	496	0.02				0.00 (0.00, 0.00)	448	0.01			
Included in SEDSS catchment area	-0.69 (-1.41, 0.02)	495	0.02	-0.78 (-1.18, -0.38)			-0.08 (-0.18, 0.03)	446	0.01	-0.18 (-0.22, -0.13)		

The final model shows covariates included in the final analysis.

*Non-linear terms; estimated degrees of freedom (edf) are provided, and spline terms are shown in S1 Fig

Earlier outbreak midpoints were associated with northern latitude (log HR: -0.30, 95% CI: -0.32, -0.29) (Table 2), increased mean monthly temperature (log HR: -0.04, 95% CI: -0.05, -0.03), residence in Ponce (where SEDSS operates) (log HR: -0.18, 95% CI:-0.22, -0.13) and eastern longitude (spline edf = 6.5) (S2B Fig). The longitude spline indicated that earlier midpoints were most likely to occur in the longitudinal vicinity of San Juan. There was no association with timing of the first case, the total number of cases, or proximity to cities.

**Fig 4:**
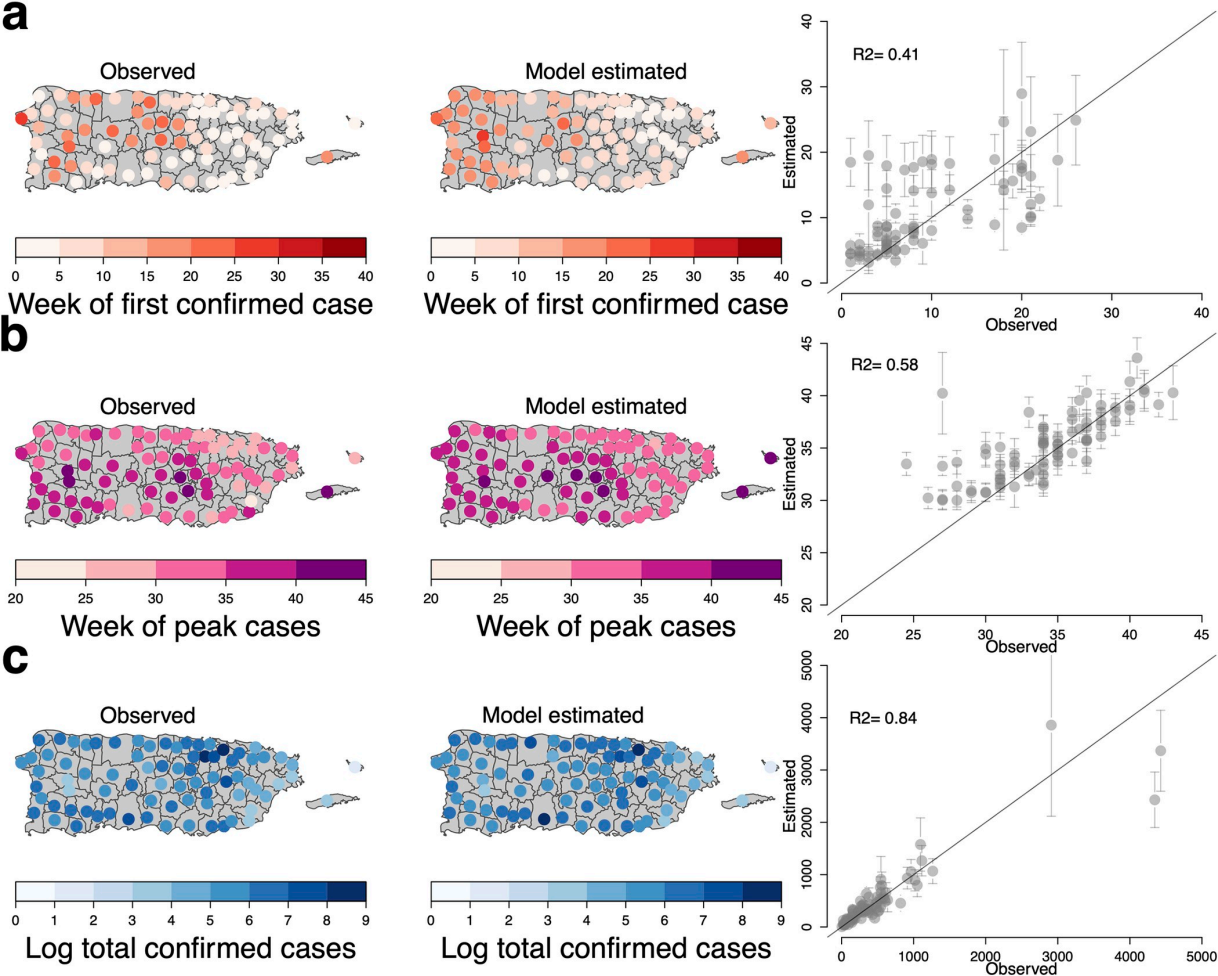
Observed and model estimated week of first confirmed Zika virus disease case, outbreak midpoint, and total confirmed cases, Puerto Rico. A: the color of the points in each municipality refers to the observed and model estimated week of first confirmed Zika virus disease case. B: the color of the points in each municipality refers to the observed and model week of the midpoint of confirmed Zika virus disease cases. C: the color of the points in each municipality refers to the observed and model estimated total confirmed cases. Grey diagonal line refers to perfect linear relationship of the observed and estimated weeks and cases (i.e. R^2^ = 1).

We compared the estimated first week and midpoint of the outbreak to those observed for each municipality to examine model fit (Fig 4A and 4B). The first confirmed ZIKV disease cases occurred mainly in the northeastern part of the island, followed by coastal areas, and eventually the western interior. The model largely replicated this pattern with the earliest cases estimated to occur in the eastern region of the island and several more populous municipalities in the West, though it overestimated the time to the first confirmed case in many western coastal municipalities (R^2^ = 0.41). Most municipalities reached the midpoint of the outbreak between the 31^st^ and 36^th^ weeks with a clear east-to-west pattern (Fig 4B). Both this range of dates and the general spread pattern were captured by the model (R^2^ = 0.58); however, some of the more densely populated areas (e.g., San Juan and Ponce) reached the midpoint earlier than anticipated by the model, along with some municipalities with smaller populations in the southeast and Culebra.

Increased log total of incident ZIKV disease cases was associated with lower mean precipitation and inclusion in the SEDSS catchment area (Table 3). Eastern longitude had a nonlinear association (spline edf = 6.9) with decreased number of incident cases (S2C Fig). Municipality-level socioeconomic factors such as average number of people per household and median household income that were significant in univariable analyses were not associated with total incident cases in multivariate analyses.

**Table 3:** Socioeconomic, climatic, and spatial factors associated with and total incident Zika virus disease cases, Puerto Rico, November 1, 2015–December 31, 2016.

Model outcome	Total incident Zika virus disease cases					
Model type	Univariate models			Final model		
Variable	Estimate (95% CI)	AIC	R^2^	Estimate (95% CI)	AIC	R^2^
Percent (%) employed	-0.02 (-0.04, 0.00)	1052	0.80		988	0.84
Average time to work (in minutes)	0.014 (-0.001, 0.27)	1051	0.77			
Median household income (in US dollars)^†^	0.00 (0.00, 0.00)	1053	0.79			
Median household age (in years)^†^	-0.03 (-0.11, 0.04)	1055	0.79			
2016 population density	0.001 (0.001, 0.001)	1055	0.78			
Longitude	-0.73 (-1.04, -0.45)	1038	0.77	* 6.9 edf		
Latitude	-1.13 (-2.11, -0.14)	1049	0.79			
Time (in minutes) to city	-0.017 (-0.019, -0.015)	1053	0.79			
Mean temperature (C)	0.23 (0.17, 0.29)	1055	0.79			
Mean precipitation (mm)	-0.002 (-0.002, -0.001)	1033		-0.001 (-0.001, 0.001)		
Inclusion in SEDSS catchment area	1.00 (0.00, 1.34)	1051	0.8	0.10 (-0.42, 0.63)		

* The final model shows covariates included in the final analysis. Non-linear terms; estimated degrees of freedom (edf) are provided, and spline terms are shown in S1 Fig

†Obtained from the 2015 American Community Survey (reference #34)

**Fig 5:**
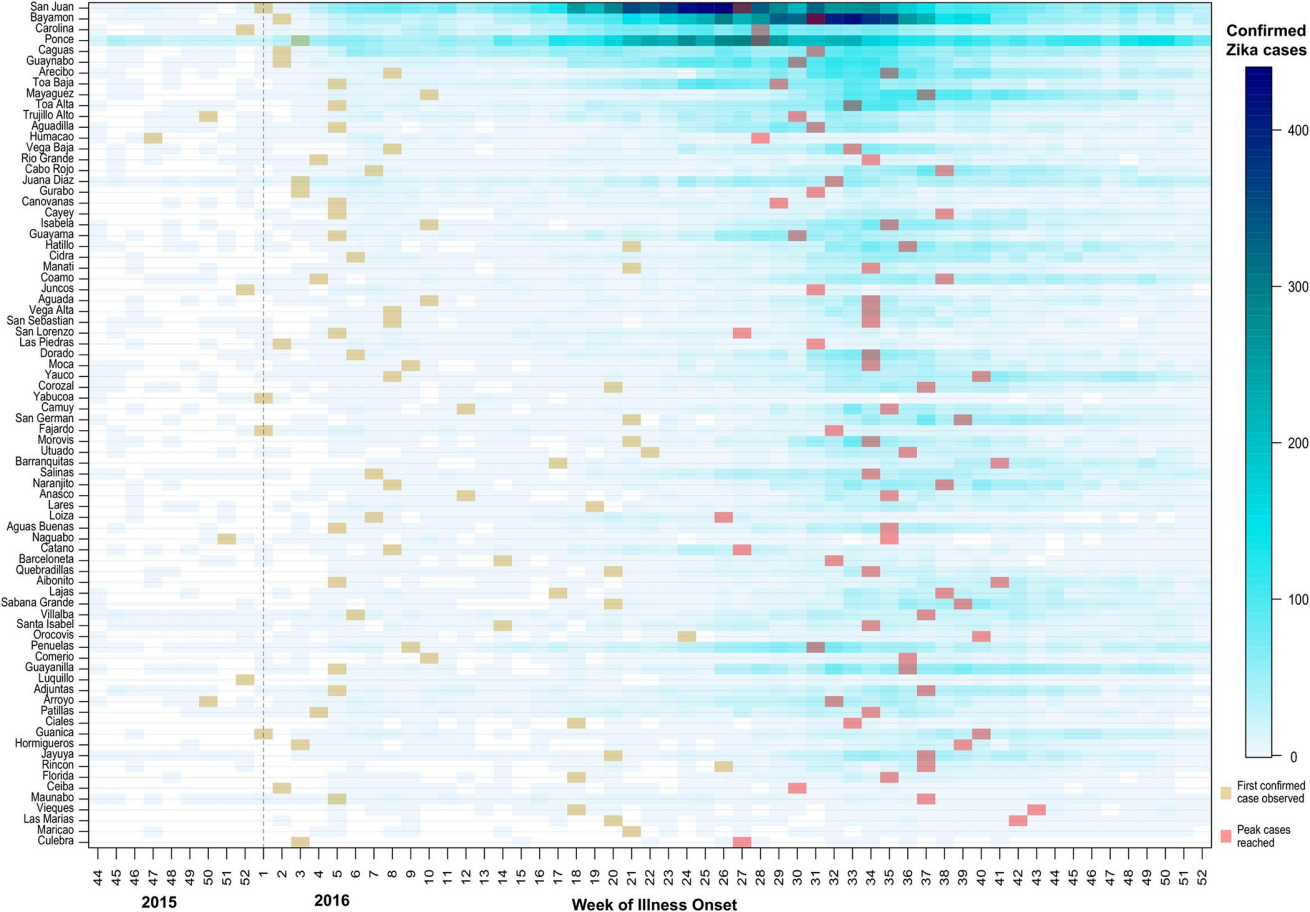
Weekly number of confirmed Zika virus disease cases by municipality, Puerto Rico, November 1, 2015–December 31, 2016 (ordered from largest to smallest municipal population, top to bottom). The points indicate the week of the first confirmed Zika virus disease case (yellow) and the week in which the midpoint of confirmed Zika virus disease cases occurred (red).

We compared the number of incident ZIKV disease cases to the model estimated cases for each municipality to assess model fit (Fig 4C). San Juan, Ponce, and Bayamon had the highest number of confirmed ZIKV disease cases (Fig 5). The fewest confirmed ZIKV disease cases occurred along eastern coastal region, the interior western region, and smaller island municipalities. Our model estimated a similar number of confirmed ZIKV disease cases (R^2^ = 0.84), though it underestimated the number of cases for San Juan and Bayamon.

## Discussion

Following detection of ZIKV transmission in the Americas in 2015, surveillance for arboviral diseases in Puerto Rico was adapted to detect ZIKV disease cases. These adaptations, together with strong support from local and federal public health partners [38], enabled testing for ZIKV infection among >71,000 suspected cases, which in turn enabled analysis of detailed epidemiologic and spatiotemporal characteristics of the epidemic. Use of the Trioplex RT-PCR assay facilitated this analysis by enabling simultaneous diagnostic testing of most patients reported with suspected arboviral disease for evidence of ZIKV, DENV, and CHIKV infection.

During the 2016 Zika epidemic in Puerto Rico, 1% of residents of Puerto Rico were reported to public health authorities as having suspected ZIKV disease and had laboratory evidence of ZIKV infection. This rate is two-fold lower than that reported from Cabo Verde [39] and 10-fold higher than in the Dominican Republic [40], but may not reflect true differences in the magnitude of the epidemic. Rather, availability of surveillance and diagnostic resources in Puerto Rico may have resulted in increased case detection compared to other jurisdictions. In Puerto Rico and elsewhere, ZIKV disease case detection is hindered by many ill individuals not seeking medical care and some clinically-apparent cases not being reported to health authorities. One report from Puerto Rico found that although over half of individuals with ZIKV disease sought medical care, only one-in-five were reported to public health authorities [41]. The number of identified ZIKV disease cases represents only a fraction of the total number of individuals infected with ZIKV, as reflected by estimates of infection rates among adult blood donors from Puerto Rico [42].

As previously reported from Puerto Rico [43] and consistent with reports from other jurisdictions [3, 40, 44, 45], adult females were over-represented among ZIKV disease patients. Although sexual transmission cannot be discounted as playing a role in this imbalance [46], a more likely explanation may be sex- and age-specific differences in frequency of developing symptomatic ZIKV infection [41, 47]. Further investigation is needed to elucidate the specific factors and the magnitude of their possible effect on the pathophysiology of ZIKV infection, such as quantitation of *in vivo* levels of various hormones [48] among individuals with a range of ZIKV infection outcomes.

Despite routine testing for both dengue and chikungunya, stark decreases in cases of both diseases were observed as ZIKV spread across the island, in contrast to observations from Brazil [49]. Whereas the decrease in chikungunya cases may be the result of a waning chikungunya epidemic co-incident to increasing ZIKV transmission, many jurisdictions in the Americas observed a decrease in dengue cases soon after detection of local ZIKV transmission [49, 50]. Contemporaneous studies from Brazil and Nicaragua demonstrated evidence of short-term protection from ZIKV infection or development of ZIKV disease associated with recent DENV infection [47, 50]. Initial analyses demonstrated a low frequency of cross-neutralization following ZIKV infection, suggesting that ZIKV does not elicit long-lasting cross-protective antibodies against DENV [51, 52]. Both protection from and enhancement of dengue severity has been associated with prior exposure to other flaviviruses [53, 54].

After examining demographic, spatial, and climatic factors associated with the geographic progression of the 2016 Zika epidemic in Puerto Rico, we found that increased population size and eastern longitude were associated with earlier detection of confirmed ZIKV disease cases. Residing in a municipality located a short distance to a city was associated with earlier detection of confirmed cases, suggesting that larger cities such as San Juan and Ponce and surrounding municipalities likely contributed to rapid spread of ZIKV across the island. In contrast, more isolated municipalities with smaller populations had later initial confirmed infections. The significant effect of longitude persisted despite the inclusion of travel time, potentially reflecting the importance of the San Juan-Carolina-Caguas metropolitan area, which encompasses approximately two-thirds of the population of Puerto Rico, and all of the eastern Puerto Rico municipalities, where initial cases were detected by the end of January 2016.

We found that northern latitude and increased mean monthly temperature were associated with earlier midpoints in local outbreaks. The northern latitude association and a weaker association with eastern longitude are likely due to the large municipalities clustered around San Juan, where the earliest midpoints occurred. However, the midpoints of the outbreak were not related to either the timing of the first confirmed cases nor travel time to large cities, indicating that other factors like temperature may have been more important to the speed of outbreak progression or the timing of peak transmission. The association with temperature implies that intense local transmission may have been delayed in the cooler, centrally-located mountainous municipalities. This is likely related to increased mosquito reproduction, survival, and viral replication within *Ae*. *aegypti* mosquitoes at higher temperatures [55], and agrees with previous findings on the importance of temperature to DENV transmission dynamics among the municipalities of Puerto Rico [56]. Comparing model predictions to observations, midpoints were observed earlier than expected in some areas with larger populations and also some with very small populations. While the multivariate model did not include population size, it is possible that there is a more complex effect that was not captured due to the limited number of municipalities.

At the municipality-level, socioeconomic characteristics (e.g., frequency of employment, travel time to place to work) were associated with the overall incidence of ZIKV disease cases in univariate analyses, but not in multivariate analyses. It is likely that these findings mask smaller scale associations between infection risk and lower socio-economic status as have been shown for DENV infection [57, 58]. Previous reports also suggest that lower regional gross domestic product is a strong predictor of ZIKV transmission [59]; within Puerto Rico, we did not observe that association either, possibly due to higher homogeneity of socioeconomic factors. For example, the median household income across the 78 municipalities was $16,852 (IQR: $15,075, $19,065) with larger cities having slightly higher median household incomes (e.g., $21,243 in San Juan, $24,430 in Bayamon and $16,318 in Ponce). Instead, spatial and climatic effects appeared to play a more important role. Interestingly, average temperature was associated with the peak week of ZIKV transmission, but not incidence. Rather, higher incidence was associated with decreased mean precipitation. This association may reflect increased habitat created by water storage in drier municipalities.

Although the surveillance activities described herein enabled systematic identification of patients with ZIKV disease, our findings remain subject to several limitations. First, our analyses relied on surveillance and laboratory testing, which are strong but imperfect indicators of the underlying infection dynamics. Surveillance efforts also vary: municipalities in the sentinel surveillance system catchment areas had higher incidence of ZIKV disease, reflecting additional efforts to improve the sensitivity of detection and reporting of cases from these municipalities. Second, the analyses were only intended to identify characteristics associated with the spatiotemporal dynamics of the ZIKV epidemic in Puerto Rico between municipalities, not to establish causal effects of these characteristics, and our descriptive analysis was specific to this setting. Therefore, although the findings are not generalizable to other locations, they may aid in identifying similar trends in other areas with arbovirus introductions. Third, we examined several factor combinations in our analysis, but did not adjust for multiple comparisons, which implies that some of the reported associations may have occurred by chance. In addition, some cases of ZIKV disease, dengue, and chikungunya were defined as such solely by detection of IgM antibody. Therefore, serologic cross reactivity between flaviviruses may have led to misclassification of some cases [27]; however, because DENV transmission was quite low in 2016, few dengue cases would be expected to have been misclassified as ZIKV disease cases. Last, as the variables utilized for our analyses originated from the clinician(s) managing the patient or the patient themselves, some variables were unable to be confirmed (e.g., signs and symptoms, status of pregnancy) or were not provided. Such missing or inaccurate data may have affected the results of the reported trends.

In summary, Puerto Rico experienced a large ZIKV outbreak in 2016 in which ~1% of residents were reported to public health authorities and had evidence of ZIKV infection. Fine scale analysis of the epidemiologic and spatiotemporal characteristics demonstrated that, in contrast to prior epidemics of dengue and chikungunya that began in the San Juan metropolitan area [11, 12], ZIKV transmission was first detected in eastern Puerto Rico in late 2015 and rapidly spread to all municipalities of the island during 2016. As expected, geography, climate, community characteristics, and human factors all influenced the observed trends of ZIKV disease. Spread was likely driven by movement between and from large cities, while outbreak progression and magnitude were more closely related to climatic factors. These findings highlight the complexity of the ZIKV epidemic and components that will likely impact future arboviral epidemics.

## Supporting information

S1 Checklist STROBE checklist. (DOC)

S1 Table Evaluation of time-to event model distributions and fit. (DOCX)

S1 Fig Summary of diagnostic testing algorithm employed in Puerto Rico during the 2015–2016 Zika virus outbreak. Refer to Methods for references of diagnostic tests utilized. Epidemiologic case classifications shown are those used in the present analysis, which are not necessarily equivalent to the interpretation of the diagnostic test results(s) sent to medical providers.
(DOCX)

S2 Fig Adjusted log hazard ratios of the nonlinear relationships between: A) travel time to the nearest city (estimated degrees of freedom [e.d.f.] of 2.0) and time to the first confirmed case of Zika virus disease; B) longitude and time to peak week of cases (e.d.f of 6.7), and C) longitude and the log cumulative incidence (e.d.f. of 6.9) (95% confidence intervals shown in grey).
(DOCX)
