# Supplementary material for: Epidemiologic and spatiotemporal trends of Zika Virus disease during the 2016 epidemic in Puerto Rico
Source: PLoS Negl Trop Dis. 2020 Sep 21;14(9):e0008532. doi: 10.1371/journal.pntd.0008532 (PMC7529257; doi:10.1371/journal.pntd.0008532)
Supplement: S1 Table — (DOCX) [file pntd.0008532.s002.docx]

**Supporting Table 1.** Evaluation of time-to event model distributions and fit

| **Model*** | **Distribution** | **AIC** |
| --- | --- | --- |
| **Time to first confirmed case** | Weibull | 496 |
|  | Log-Normal | 499 |
|  | Gaussian | 526 |
|  | Exponential | 505 |
| **Time to midpoint of the outbreak** | Weibull | 446 |
|  | Log-Normal | 447 |
|  | Gaussian | 445 |
|  | Exponential | 709 |

*Estimates use intercept-models under different model distributions
