# Supplementary material for: Epidemiologic and spatiotemporal trends of Zika Virus disease during the 2016 epidemic in Puerto Rico
Source: PLoS Negl Trop Dis. 2020 Sep 21;14(9):e0008532. doi: 10.1371/journal.pntd.0008532 (PMC7529257; doi:10.1371/journal.pntd.0008532)
Supplement: S1 Fig — Refer to Methods for references of diagnostic tests utilized. Epidemiologic case classifications shown are those used in the present analysis, which are not necessarily equivalent to the interpretation of the diagnostic test results(s) sent to medical providers. (DOCX) [file pntd.0008532.s003.docx]

**Supporting Figure 1.** Summary of diagnostic testing algorithm employed in Puerto Rico during the 2015–2016 Zika virus outbreak. Refer to Methods for references of diagnostic tests utilized. Epidemiologic case classifications shown are those used in the present analysis, which are not necessarily equivalent to the interpretation of the diagnostic test results(s) sent to medical providers.

**
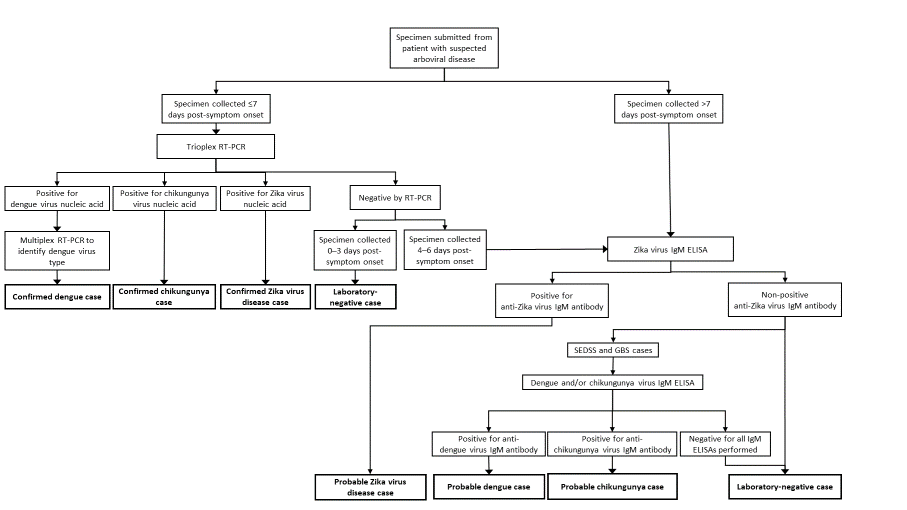
**
