# Supplementary material for: Epidemiologic and spatiotemporal trends of Zika Virus disease during the 2016 epidemic in Puerto Rico
Source: PLoS Negl Trop Dis. 2020 Sep 21;14(9):e0008532. doi: 10.1371/journal.pntd.0008532 (PMC7529257; doi:10.1371/journal.pntd.0008532)
Supplement: S2 Fig — Adjusted log hazard ratios of the nonlinear relationships between: A) travel time to the nearest city (estimated degrees of freedom [e.d.f.] of 2.0) and time to the first confirmed case of Zika virus disease; B) longitude and time to peak week of cases (e.d.f of 6.7), and C) longitude and the log cumulative incidence (e.d.f. of 6.9) (95% confidence intervals shown in grey). (DOCX) [file pntd.0008532.s004.docx]

**Supporting Figure 2.** Adjusted log hazard ratios of the nonlinear relationships between: A) travel time to the nearest city (estimated degrees of freedom [e.d.f.] of 2.0) and time to the first confirmed case of Zika virus disease; B) longitude and time to peak week of cases (e.d.f of 6.7), and C) longitude and the log cumulative incidence (e.d.f. of 6.9) (95% confidence intervals shown in grey).
